# WRKY transcription factors participate in abiotic stress responses mediated by sugar metabolism

**DOI:** 10.3389/fpls.2025.1646357

**Published:** 2025-08-07

**Authors:** XueYi Zhang, WanXia Liu, YiAn Yin, Jia Zheng, JianAn Li, XiaoFeng Tan, LingLi Wu

**Affiliations:** ^1^ College of Forestry, Central South University of Forestry and Technology, Changsha, China; ^2^ Key Laboratory of Cultivation and Protection for Non-wood Forest Trees, Ministry of Education, Central South University of Forestry and Technology, Changsha, China; ^3^ Key Laboratory of Non-wood Forest Products of State Forestry Administration, Central South University of Forestry and Technology, Changsha, China; ^4^ Engineering Technology Research Center of Southern Hilly and Mountainous Ecological Non-Wood Forest Industry of Hunan Province, Central South University of Forestry and Technology, Changsha, China; ^5^ Lutou National Station for Scientific Observation and Research of Forest Ecosystem in Hunan Province Changsha, Changsha, China

**Keywords:** WRKY transcription factor, sugar metabolism, abiotic stress, regulation mechanism, plant growth and development

## Abstract

Plant abiotic stress refers to the unfavorable effects on plants caused by any abiotic factors in a specific environment, such as drought, high temperature, low temperature, etc., which cause disruption of plant physiology and metabolism, and seriously affect the growth and yield of plants. Mounting evidence demonstrates that WRKY transcription factors modulate plant abiotic stress responses by regulating sugar metabolic pathways. Sugar metabolism pathway plays an essential role in plant stress resistance, and WRKY transcription factors, as an important class of regulatory factors, have attracted wide attention for their mechanism of action in abiotic stress. Therefore, this review primarily aims to analyze the structure and classification of WRKY transcription factors, summarize the research progress on how WRKY transcription factors themselves respond to stress, and how they participate in regulating plant stress responses through sugar metabolism pathways. Through in-depth investigation of the relationship between WRKY transcription factors and sugar metabolic pathways we uncovered novel abiotic stress-related gene regulatory networks providing theoretical basis and practical guidance for genetic improvement of plants under abiotic stress.

## 1 Introduction

Plant abiotic stress has become one of the key constraints to agricultural production and food security. Among them, drought, cold, and salinity stress are primary abiotic factors that impair plant growth and development and constrain their geographic distribution. These stresses often cause similar effects on plants, such as disrupting cellular osmotic balance, damaging cell membrane structures, and impairing antioxidant defense systems (Qu et al., 2019). To survive adverse environmental conditions, plants have evolved intricate signaling and gene regulatory pathways. These sophisticated mechanisms enable adaptation and mitigation against the detrimental impacts of abiotic stresses. Sugar metabolism, as one of these pathways, plays a crucial role in the process by which plants resist abiotic stress (Qin et al., 2018; Ma et al., 2019; Yoon et al., 2020). Sugars not only provide energy and carbon sources but also participate in signal transduction and the regulation of physiological processes. Although a large number of studies have shown that sugar metabolism is extensively involved in plant stress, relatively few studies have been conducted on how sugar mediates stress mechanisms.

Transcription factors, as key components of signal transduction, play the role of “molecular switches” in the transcriptional regulatory networks of abiotic stress responses. WRKY transcription factors (TFs), a unique class of proteins in higher plants, are widely involved in regulating various physiological and metabolic pathways. They are capable of highly specific recognition and binding to cis-acting elements called W-box on DNA sequences. This binding directly regulates the transcription levels of target genes, including self and other stress-related genes, and thus plays a key role in plant response to abiotic stresses. In addition, WRKY TFs can also bind to cis-acting elements in the promoter regions of sugar metabolism genes and regulate the expression of sugar metabolism-related genes (Sun et al., 2003; Govardhana and Kumudini, 2020; Goyal et al., 2020). Through regulation of the sugar metabolism pathway, they mediate responses to stress, thus improving plant tolerance to abiotic stresses and injury, and mitigating the damage inflicted by stress on plants (Wu et al., 2020).

Therefore, this paper mainly analyzed the structure and classification of WRKY transcription factors, summarized the research results and reviewed the regulatory mechanisms of WRKY transcription factors themselves in response to abiotic stresses as well as their involvement in abiotic stresses in plants through sugar metabolism pathways, with a view to providing a theoretical basis for the genetic improvement of plant stress tolerance, and providing a technological safeguard for the enhancement of agricultural production and the assurance of food safety.

## 2 Structural characteristics and classification of WRKY TFs

WRKY TFs are among the largest families of transcription factors in higher plants and are designated as the “central regulators” of the abiotic stress response. The first WRKY TFs, *SPF1*, was originally identified and isolated from *Ipomoea batatas* (Ishiguro and Nakamura, 1994). Subsequent studies have characterized numerous WRKY TFs across diverse plant species, including. *Arabidopsis* (Glöckner et al., 2002), *Oryza sativa* (Kim et al., 2000), *soybean* (Schmutz et al., 2010), and *Hordeum vulgare* (Mangelsen et al., 2008). Comprehensive research has elucidated the extensive membership and multifaceted regulatory mechanisms characterizing the WRKY transcription factor family. By constructing complex signaling networks, WRKY TFs play a crucial role in plant growth, development, and stress responses (ülker and Somssich, 2004; Rushton et al., 2010).

**Figure 1:**
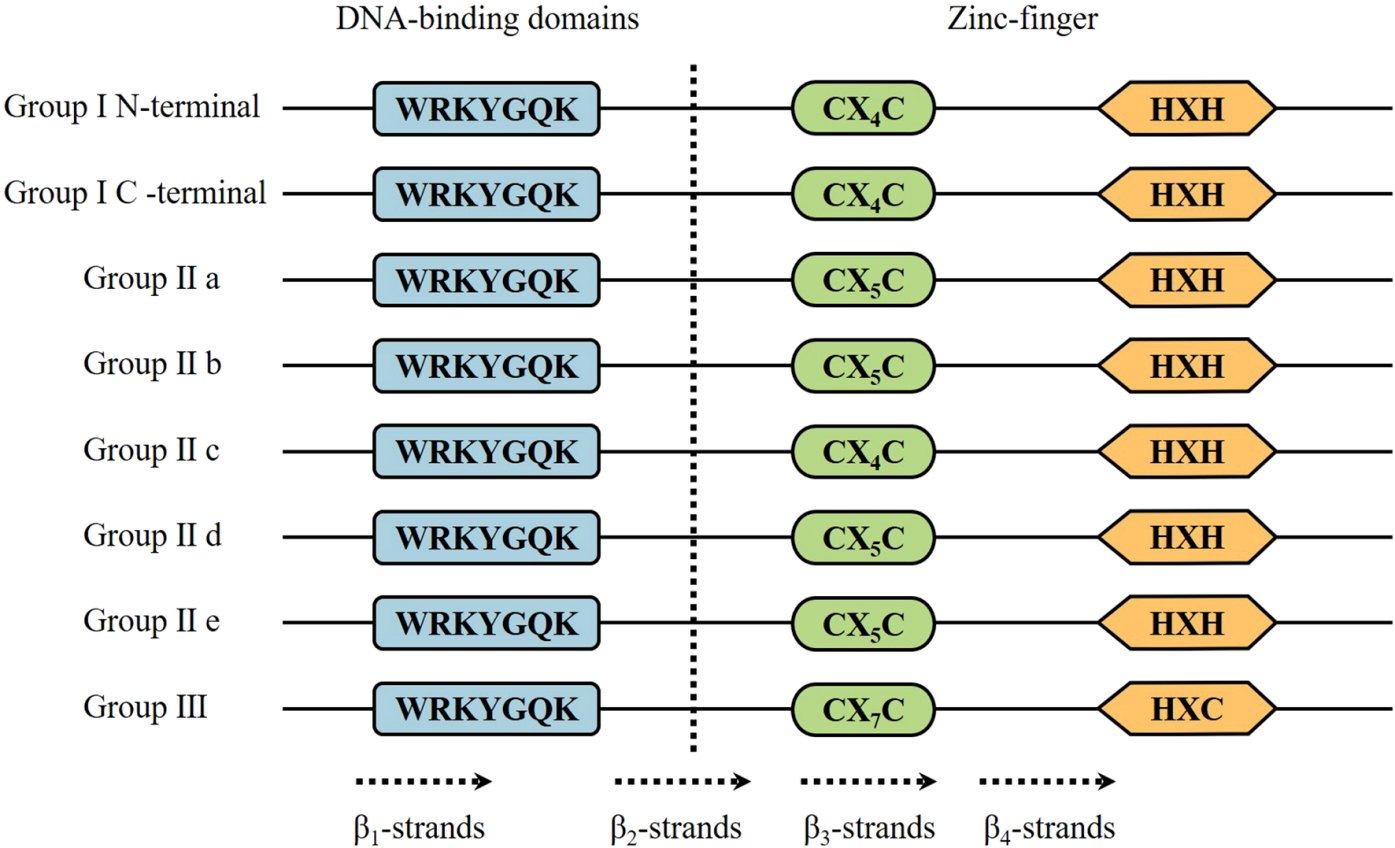
Domain structures of different WRKY subfamilies in higher plants. The WRKY motif, the cysteines, and the histidines that form the zinc finger are shown in boxes. I N and I C denote the N-terminal and C-terminal domains from Group I WRKY proteins, respectively. The 4 β-strands are shown with dashed arrows.

WRKY TFs derive their nomenclature from the characteristic WRKY domain, defined by the highly conserved WRKYGQK motif (Wu et al., 2020). This family of proteins is characterized by the fact that all family members contain at least one WRKY structural domain consisting of about 60 highly conserved amino acids, the N-terminal end of which contains the highly conserved WRKYGQK heptapeptide sequence, and the C-terminal end of which has a zinc-finger motif of either the C_2_H_2_ or the C_2_HC type (Wang et al., 2014b). WRKY TFs specifically recognize W-box cis-elements (A/TAACCA; C/TAACG/TG) in target gene promoters, thereby modulating transcription. Depending on the number of conserved WRKY domains and the type of zinc finger structure, WRKY transcription factors are usually divided into three families: family I contains two WRKY domains and two C_2_H_2_ zinc finger structures, family II contains one WRKY domain and one C_2_H_2_ zinc finger structure, and family III contains one WRKY domain and one C_2_H_2_ zinc finger structure. Family II is subdivided into five subfamilies: a, b, c, d, and e. Family II WRKY proteins are involved in the regulation of plant growth and development, such as senescence, seed dormancy, and germination; they are also involved in plant responses to drought, salt stress, and cold damage (Rushton et al., 2012). WRKYs cannot form homologous or heterodimers if they do not have LZ (leucine zipper) motifs (Narusaka et al., 2016). In addition to the above structural domains, WRKY transcription factor families have many other structures, such as kinase domains, glutamine rich regions, proline rich regions, nuclear localization signals, and so on. The existence of these structural domains makes it possible for these WRKY proteins to regulate the expression of target genes through the formation of homodimers or heterodimers by protein-protein interactions (Zhang and Wang, 2005; Agarwal et al., 2011) (**Figure 1**).

## 3 WRKY TFs involved in abiotic stress responses

**Figure 2:**
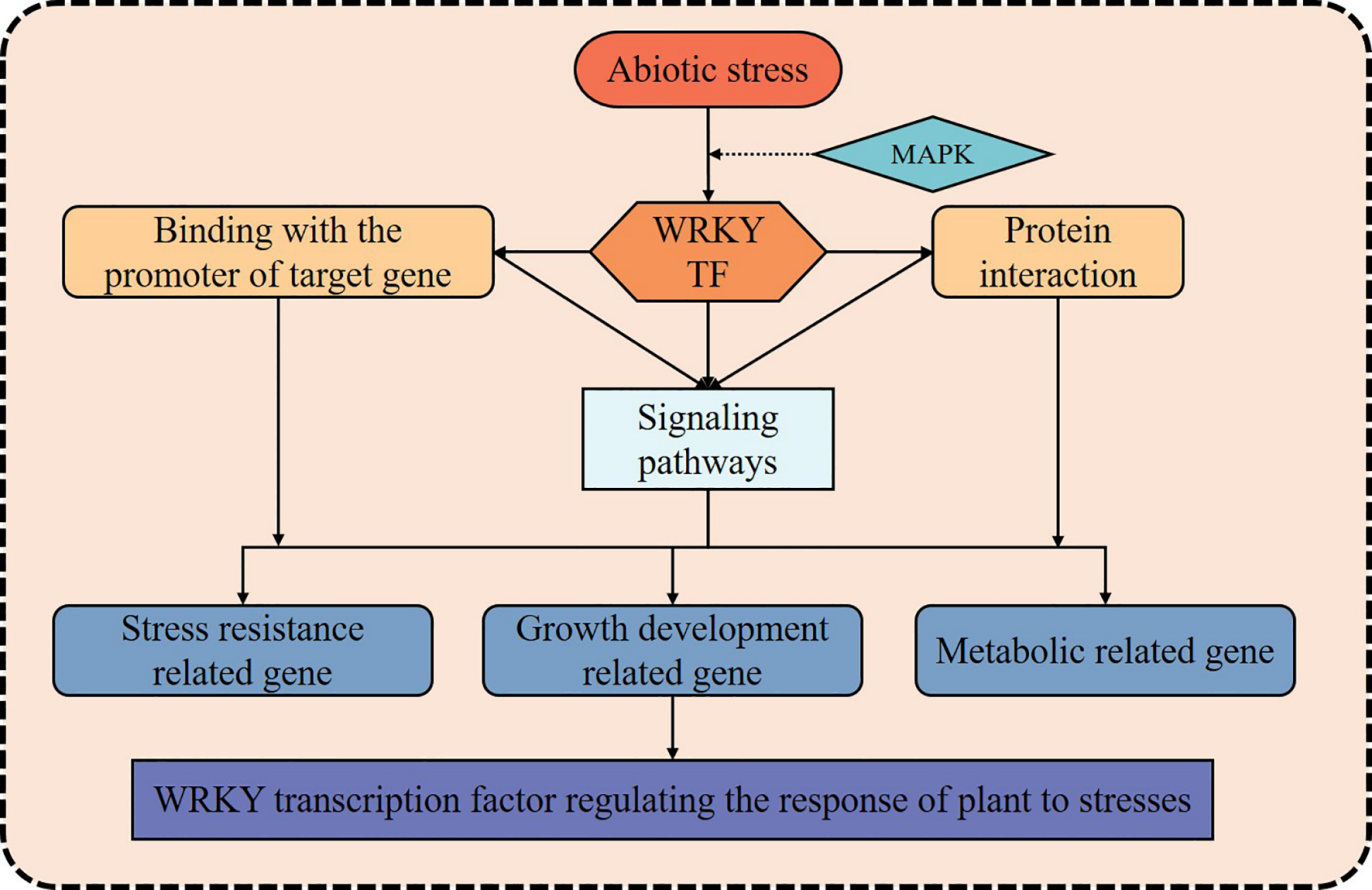
The diagram of WRKY transcription factor regulating stress responses in plants. The solid black arrows indicate that WRKYs regulating plant stress response pathway; The dotted black arrow indicates that WRKY transcription factors might be activated by the MAPK cascade and thus participates in the regulation of stress response.

In recent years, as climate change and extreme weather events have increased, the impact of abiotic stress on crop production has become more pronounced, resulting in slowed growth, deteriorating quality, and reduced yields (Hrmova and Hussain, 2021; Khoso et al., 2022). Consequently, over the course of long-term natural selection and evolutionary processes, plants have developed a complex and finely tuned regulatory network that enables them to detect and respond effectively to various environmental stresses (Su et al., 2023). Facing abiotic stress, WRKY TFs could dynamically modulate downstream gene expression, either enhancing transcriptional activation or imposing repression, directly regulating the expression of genes involved in stress response, or participate in other signaling pathways and regulatory networks to manage the stress response (**Figure 2**). This activation of defense mechanisms helps enhance crop resilience against abiotic stress (Ma and Hu, 2024). Given that extensive and in-depth studies and reviews have already been conducted on WRKY TFs’ roles in responding to abiotic stress (**Table 1**), this paper provides only a concise summary of the key findings.

**Table 1:** WRKY TFs involved in abiotic stress responses in plants.

No.	*Gene*	*Species*	Stress responses	References
1	*MaWRKY70*	*Musa acuminata*	Tolerance to cold	(Lin et al., 2024)
2	*OsWRKY63*	*Oryza sativa*	Tolerance to cold	(Zhang et al., 2022b)
3	*OsWRKY76*	*Oryza sativa*	Tolerance to cold	(Naoki et al., 2013)
4	*OsWRKY74*	*Oryza sativa*	Tolerance to phosphate (Pi) starvation	(Dai et al., 2016)
5	*CsWRKY19*	*Camellia sinensis*	Tolerance to cold	(Guo et al., 2024)
6	*VpWRKY1*	*Vitis pseudo-reticulata*	Tolerance to salt and cold	(Li et al., 2010)
7	*VpWRKY2*	*Vitis pseudo-reticulata*	Tolerance to salt and cold	(Li et al., 2010)
8	*GmWRKY13*	*Glycine max*	Tolerance to drought and cold	(Zhou et al., 2008)
9	*GmWRKY21*	*Glycine max*	Tolerance to drought and cold	(Zhou et al., 2008)
10	*GmWRKY54*	*Glycine max*	Tolerance to drought and cold	(Zhou et al., 2008)
11	*CsWRKY51*	*Cucumis sativus*	Tolerance to cold	(Lu et al., 2025)
12	*CwWRKY65*	*Camellia weiningensis*	Tolerance to cold	(Xu and Xu, 2024)
13	*VbWRKY32*	*Verbena bonariensis*	Tolerance to cold	(Wang et al., 2020)
14	*VvWRKY24*	*Vitis vinifera*	Tolerance to cold	(Wang et al., 2014a)
15	*AtWRKY34*	*Arabidopsis*	Tolerance to cold	(Zou et al., 2010)
16	*PoWRKY69*	*Paeonia ostii*	Tolerance to drought	(Luan et al., 2024)
17	*MbWRKY46*	*Malus baccata*	Tolerance to drought and cold	(Liu et al., 2023)
18	*ChaWRKY40*	*Corylus avellana*	Enhances Drought Tolerance	(Zhang et al., 2024a)
19	*PwuWRKY48*	*Populus wulianensis*	Enhances Drought Tolerance	(Wang et al., 2024)
20	*EjWRKY17*	*Eriobotrya japonica*	Enhances Drought Tolerance	(Wang et al., 2021)
21	*IgWRKY32*	*Iris germanica*	Enhances Drought Tolerance	(Zhang et al., 2022a)
22	*IgWRKY50*	*Iris germanica*	Enhances Drought Tolerance	(Zhang et al., 2022a)
23	*PtWRKY33*	*Populus trichocarpa*	Tolerance to drought and salt	(Yang et al., 2023)
24	*ZmWRKY40*	*Zea mays*	Enhances Drought Tolerance	(Wang et al., 2018)
25	*IlWRKY70*	*Iris laevigata*	Tolerance to drought and salt	(Shi et al., 2023)
26	*BnWRKY49*	*Boehmaeria nivea*	Enhances Drought Tolerance	(Bao et al., 2024)
27	*AfWRKY2*	*Amorpha fruticosa*	Enhances Drought Tolerance	(Li et al., 2023)
28	*StWRKY6*	*Solanum tuberosum*	Tolerance to cadmium (Cd)	(He et al., 2023)
29	*OsWRKY54*	*Oryza sativa*	Tolerance to salt	(Huang et al., 2022a)
30	*VuWRKY21*	*Vigna unguiculata*	Tolerance to salt	(Crispim et al., 2023)
31	*VuWRKY87*	*Vigna unguiculata*	Tolerance to salt	(Crispim et al., 2023)
32	*ZjWRKY18*	*Ziziphus jujuba*	Tolerance to salt	(Wen et al., 2023)
33	*AhWRKY75*	*Arachis hypogaea*	Tolerance to salt	(Zhu et al., 2021)
34	*GmWRKY16*	*Glycine max*	Tolerance to drought and salt	(Ma et al., 2019)

Drought stress, as an essential abiotic stress, poses a serious threat to plant growth, development, and yield. Recent studies have identified multiple WRKY TFs as key regulators of drought tolerance. *IgWRKY50* and *IgWRKY32* in *Iris germanica*, which can enhance drought resistance in transgenic Arabidopsis by coordinated up-regulation of drought-responsive downstream genes (Zhang et al., 2022a). In *Glycine max*, *GmWRKY17* directly binds to promoters of drought-inducible genes *GmDREB1D* and *GmABA2*, activating their transcription under water deficit (Yi and Yueping, 2023). *SbWRKY30* in *Sorghum bicolor* directly activates the drought-response gene *SbRD19*, conferring improved growth and survival rates under drought stress (Yang et al., 2020).

Salt stress critically constrains plant growth and development, wherein WRKY transcription factors (TFs) execute pivotal regulatory roles. *ZmWRKY104* in *Zea mays* overexpression enhances salt tolerance via positive regulation of *ZmSOD4*, reducing ROS accumulation, MDA content, and electrolyte leakage (Yan et al., 2022).In *Gossypium hirsutum*, *GhWRKY34* confers salt tolerance by modulating selective Na^+^/K^+^ uptake and maintaining low Na^+^/K^+^ ratios in leaves/roots (Zhou et al., 2015).*GmWRKY54* in *Glycine max* activates transcription in response to salt stress by binding to the W-box elements of the promoters of the *DREB2A* and *STZ/ZAT10* genes, which are key transcription factors in the ABA-independent pathway that regulates osmoprotective substance synthesis, and *STZ/ZAT10*, which is involved in ROS scavenging and ion homeostasis maintenance (Zhou et al., 2008).

WRKY TFs also have a significant job in responding to heavy metal stresses. In *Oryza sativa*, *OsWRKY74* regulates the expression of a set of downstream genes involved in phosphorus uptake, transport, and metabolism, collectively enhancing rice tolerance to phosphorus starvation (Dai et al., 2016). Similarly, *OsWRKY72* negatively regulates lignin synthesis and accumulation by inhibiting the expression of *OsGLP8-7* (germin-like protein), thereby reducing the ability of the cell wall to retain heavy metal ions and realizing the regulation of Cd/Cu toxicity (Shangguan et al., 2024). *TaWRKY70* in *Triticum aestivum* reduces root Cd²^+^influx by re-pressing the expression of *AtNRAMP5, AtHMA3, AtYSL3*, and *AtIRT1* heavy-metal transporter genes, which act as Cd transporters, while *TaWRKY70* activates the expression of the *TaCAT5* promoter by directly binding to its W-box motif to enhance catalase activity, reduce membrane lipid peroxidation, scavenge ROS, and confer resistance to Cd stress in transgenic Arabidopsis (Jia et al., 2021).

In addition, with global climate change in recent years, both high and low temperatures have become significant agricultural meteorological disasters, severely limiting normal plant and crop development. Heat stress triggers upregulation of *WRKY25* and *WRKY26* yet downregulates *WRKY33* in *Arabidopsis thaliana*. Molecular evidence reveals that these three factors synergistically enhance thermotolerance by coordinating ethylene signaling activation with heat shock protein (HSP) pathways, leveraging functional crosstalk and complementary effects (Li et al., 2011). In *Capsicum annuum*, the transcription of *CaWRKY40* is induced by Ralstonia solanacearum and high temperature. Heat stress induces an upregulation of *CaWRKY40* expression, and its overexpression enhances heat stress tolerance, likely through the regulation of antioxidant systems and maintenance of cell membrane stability (Dang et al., 2013). Regarding low temperature, Zhang et al. (2022b) proposed a transcriptional regulatory cascade model involving *OsWRKY63–OsWRKY76–OsDREB1B*, where *OsWRKY63* acts as a transcriptional repressor by inhibiting the expression of *OsWRKY76*, thereby suppressing the activation of *OsDREB1B*, which leads to reduced cold tolerance (Zhang et al., 2022b). The expression of *VbWRKY32* was significantly increased in Verbena leaves under cold stress. Overexpression of the *VbWRKY32* gene in Verbena and comparison of the expression profiles of cold-responsive genes between overexpressed and wild-type plants under cold stress revealed that *VbWRKY32* acted as a positive regulator to enhance the cold resistance of plants by up-regulating the transcript levels of cold-responsive genes (Wang et al., 2020). In *Camellia sinensis*, *CsWRKY6, CsWRKY31, CsWRKY48* were induced to be up-regulated under 4 °C cold treatment, indicating that they acted as positive regulators involved in the regulatory pathway of *Camellia sinensis* in response to cold (Wang et al., 2019). All these studies confirmed that WRKY TFs plays an essential role in defense against abiotic stresses.

## 4 Sugar metabolism involved in abiotic stress responses

Adversities such as low temperature, high temperature, drought, and salinity usually produce water stress in plants, and osmoregulation is one of the important physiological mechanisms for plants to resist such abiotic stresses. Plants through the regulation of various physiological metabolism in body accumulation of a wide range of organic or inorganic substances to increase the concentration of cell membranes, reduce the osmotic potential, and enhance the cellular water absorption or retention capacity. Sugars are an effective carbohydrate in plant response to abiotic stress, which not only keep the cellular osmotic balance, but also participate in the signaling molecules for the perception and conduction of adversity signals, and regulate the growth and development of plants and their ability to cope with adversity (Sun et al., 2015).

### 4.1 The mechanism by which sugar responds to abiotic stress

The role played by sugar compounds in response to abiotic stresses in plants is characterized by four main aspects: First, under abiotic stress, plants accumulate sugars within cytoplasmic and vacuolar compartments. This solute accumulation elevates cytosolic concentration, modulates tissue osmotic potential, depresses freezing point, and mitigates cellular dehydration—collectively establishing a physical defense barrier against environmental adversities (Sun et al., 2015; Hu et al., 2016). Second, sugar compounds provide protective effects on biomembranes and macromolecules. Specifically, fructans demonstrate membrane-stabilizing properties by reducing the phase transition temperature between lipid gel and liquid crystalline states (Hincha et al., 2002). This phenomenon facilitates enhanced molecular interactions between sugar moieties and membrane phospholipids, thereby preserving membrane structural integrity under stress conditions. Third, plant sugar catabolism integrates bio-oxidative pathways with oxidative phosphorylation systems. This integrative mechanism can provide not only sufficient reducing power and energy for other biosynthetic processes, it also produces other protective substances that protect plant organizations from stress-induced damage (Savitch et al., 2010). Fourth, sugar compounds form complex signaling networks with other signaling molecules, regulating the expression of stress-related genes involved in metabolic activities, thereby helping plants respond to adverse environmental conditions (Smeekens et al., 2010). For instance, low-temperature stress upregulates key sucrose metabolic enzymes such as sucrose synthase (SUS) and sucrose phosphate synthase (SPS), enhancing their catalytic activity and thereby driving sucrose accumulation in plants. On the one hand, sucrose acts as an osmotic regulator, maintaining cellular osmotic potential under low temperatures and preventing cell freezing. On the other hand, as an important signaling molecule and antioxidant, sucrose can induce the expression of cold tolerance genes and key enzymes in the antioxidant system (Turhan and Ergin, 2012; Cao et al., 2014). Therefore, sugar compounds play an indispensable and crucial role in plant stress resistance, and they help plants maintain their normal physiological functions and growth status in the face of various abiotic stresses at multiple levels and through the utilization of different regulatory mechanisms.

### 4.2 The response of glucose metabolism to abiotic stress

Regarding the study of the pathway of sugar metabolism involved in stress tolerance, a large number of studies have shown that the pathway of sugar metabolism involved in stress tolerance is mainly related to the accumulation of saccharides, and that the higher the content of soluble sugars in the body of a plant, the stronger its cold tolerance will be (Ouyang et al., 2019). By accumulating soluble sugar content, plants are able to increase cellular osmotic potential, which in turn enhances cellular water retention capacity (Ouyang et al., 2019). Current research predominantly focuses on abiotic stress-induced accumulation of soluble sugars. For instance, cold acclimation elevates levels of soluble sugars like sucrose, glucose, trehalose, and raffinose in *Camellia sinensis* leaves, concomitantly increasing cold tolerance (Yue, 2015). In *Medicago sativa*, low-temperature stress induces up-regulated expression of the Galactinol Synthase (GoLS) and improves cold tolerance in *Medicago sativa* (Zhuo et al., 2013). Low temperature induces enhanced SPS activity, leading to increased sucrose accumulation (Lundmark et al., 2006; Miao et al., 2007). Similarly, overexpression of *ZmSUS1*, a key enzyme in sugar metabolism, has been found to increase drought tolerance in *Zea mays* by regulating sucrose metabolism and soluble sugar content (Xiao et al., 2024). Furthermore, seaweed extract-based bio-stimulants mitigate drought stress in *Saccharum officinarum* by enhancing leaf metabolic activity and total sugar levels (Jacomassi et al., 2022). Therefore, soluble sugars and their metabolic pathways play a pivotal role in plant responses to abiotic stress. By regulating the synthesis and metabolism of soluble sugars, plants bolster their osmotic adjustment capacity and antioxidant defenses, thereby enhancing their overall adaptability to abiotic stress.

## 5 WRKY TFs are involved in plant abiotic stress responses mediated by sugar metabolism

### 5.1 The mechanism of WRKY TFs regulate sugar metabolism

**Figure 3:**
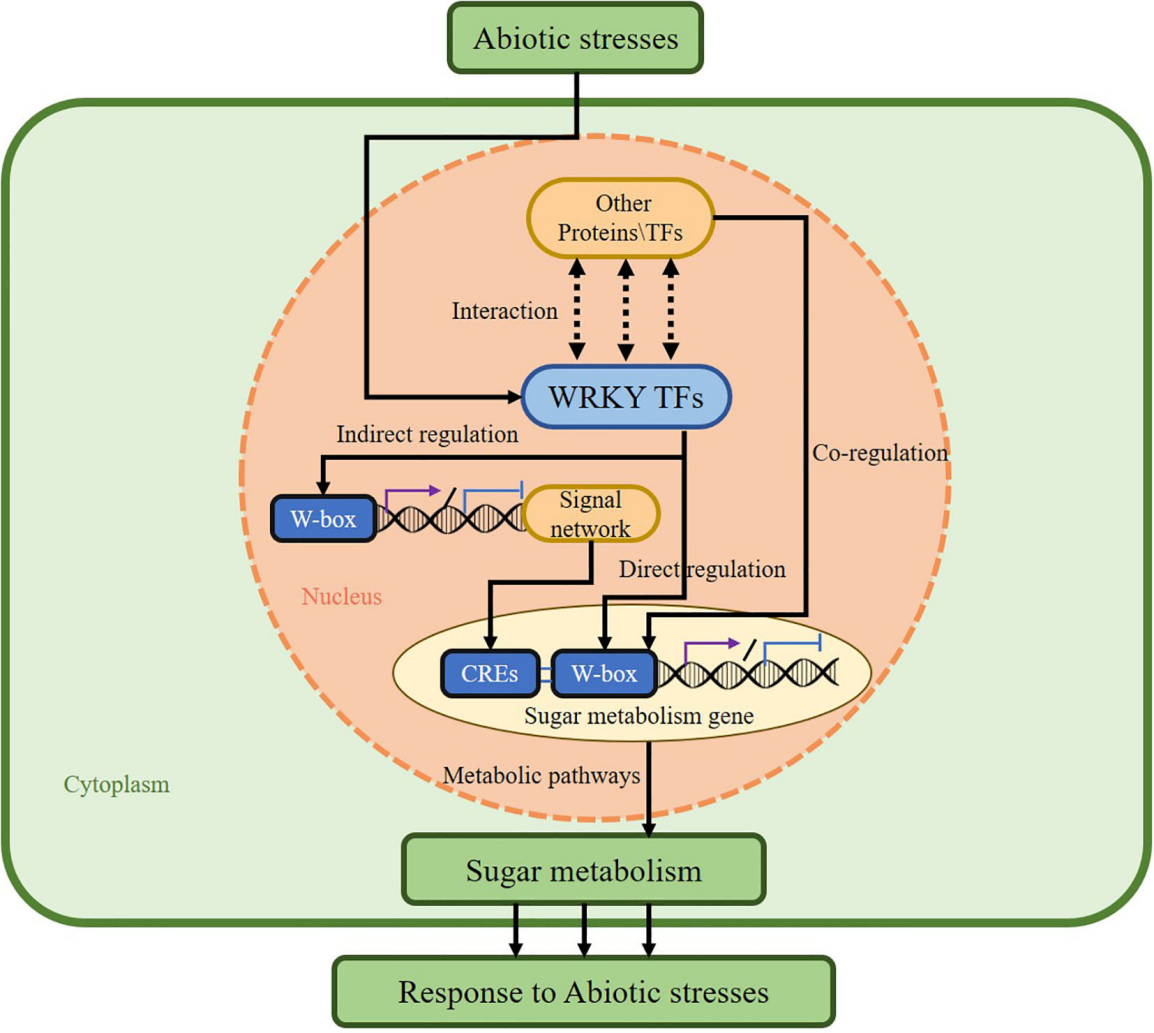
The mechanism by which WRKY TFs regulate glucose metabolism and mediate abiotic stress. We categorized the modes of regulation of WRKY transcription factors in plant sugar metabolism into three different types: Direct regulation, Indirect regulation, and Cooperative regulation.

WRKY TFs regulate plant sugar metabolism through multiple distinct pathways. First, WRKY TFs directly bind to W-box cis-elements within the promoters of sugar-metabolic genes, enabling their transcriptional regulation (Chen et al., 2019). Second, WRKY TFs indirectly modulate sugar metabolism by integrating into intricate plant signaling networks. These transcription factors are activated by diverse signals, including plant hormones, biotic stresses, and abiotic stresses, subsequently modulating sugar metabolic pathways via signaling cascades. Furthermore, WRKY TFs can form complexes with other proteins, achieving cooperative regulation of sugar metabolism through cross-family collaboration with other transcription factor families or interaction with epigenetic regulators, integrating signals and expanding the target range (Li et al., 2025). Here, this paper concludes the modes of action of WRKY TFs involved in the regulation of plant sugar metabolism and classifies them into three types: direct regulation, indirect regulation, and cooperative regulation (**Figure 3**) (Li et al., 2025). Elucidating the regulatory mechanisms of WRKY TFs in sugar metabolism will deepen our understanding of the intricate relationship between WRKY TFs and sugar metabolism.

#### 5.1.1 Direct regulation

WRKY TFs directly regulate the expression of genes encoding sugar-metabolic enzymes by binding to cis-regulatory elements (e.g., W-box or CRT/DRE motifs) within their promoters. This modulates sugar biosynthesis, degradation, and transport, ultimately enhancing plant stress tolerance. For example, *SUSIBA2*, a WRKY-like transcription factor isolated from barley, binds not merely to the W-box on the *ISO1* (isoamylase) promoter (Sun et al., 2003), and also to *SURE* (Sugar Metabolism Cis-Acting Element), thereby regulating starch synthesis. *MdWRKY126* in *Malus dasyphylla* enhances the activity of the SPS enzyme by directly binding to the promoter region of the SPS gene and upregulating its expression level (Zhang et al., 2025). In *Oryza sativa*, *OsWRKY71* binds to W-box sequences in the promoter of α-amylase genes, repressing gibberellin-induced expression of *Amy32b* (Zhang et al., 2004). Furthermore, *AtWRKY18* and *AtWRKY53* in *Arabidopsis* directly couple to the promoters of sugar-responsive genes and trigger their expression upon glucose treatment (Chen et al., 2019). In Pitaya (*Hylocereus*), WRKY TFs (e.g., *HpWRKY3, HpWRKY18, and HpWRKY44*) are associated with up-regulated expression of genes involved in betalain biosynthesis and sugar metabolism (*HpCytP450-like1, HpSS2, and HpAI2*) (Cheng, 2018).Similarly, *HpWRKY3* activates the expression of *HpINV2* and *HpSuSy1*, suggesting it may directly target their promoters to modulate transcription and regulate sucrose metabolism (Cheng, 2018; Wei et al., 2019).

#### 5.1.2 Indirect regulation

Plant sugar metabolism exhibits intricate cross-talk with hormone signaling path-ways, including ABA, JA, and SA. WRKY TFs frequently serve as pivotal integrators within hormone signal transduction networks to indirectly modulate sugar metabolism. For instance, in *Camellia sinensis*, *CsWRKY29* binds to the promoter of *CsABI5*, an ABA signaling component harboring a W-box element, and activates its expression. Subsequently, as a downstream regulator, *CsABI5* binds to *ABREs* (ABA-responsive elements) within the promoters of *CsHXK1* and *CsSUS4* (Xue et al., 2024). This establishes a “*CsWRKY29-CsABI5-HXK1/SUS4*” regulatory cascade, mediating indirect control of sugar metabolism (Xue et al., 2024). In *Oryza sativa*, *OsWRKY5* functions as a negative regulatory hub in the ABA pathway; it indirectly suppresses the activation of sugar metabolism-related genes by re-pressing *OsMYB2* expression. Following *OsWRKY5* knockout, the repression on the ABA signaling pathway is lifted, activating sugar metabolic pathways and leading to a significant increase in soluble sugar content (Lim et al., 2021). Beyond ABA signaling, *Vitis vinifera VviWRKY10* and *VviWRKY30* modulate the expression of sugar metabolism-related genes by engaging with SA and JA signaling pathways. *VviWRKY10* primarily responds to SA signals, upregulating SUS and sucrose transporter (SUT) gene expression, thereby promoting sucrose accumulation to enhance osmotic adjustment capacity (Zhou et al., 2024). In contrast, *VviWRKY30* acts through the JA signaling pathway to repress glycolysis, reducing glucose consumption and thereby prioritizing carbon allocation toward defense-related metabolism (Zhou et al., 2024). By integrating SA and JA signals, *VviWRKY10* and *VviWRKY30* cooperatively regulate powdery mildew resistance and sugar metabolism partitioning in grapevine (Zhou et al., 2024). This “bidirectional regulatory” mode exemplifies the finely tuned balance WRKY factors achieve between sugar metabolism and stress resilience (Zhou et al., 2024).

#### 5.1.3 Cooperative regulation

WRKY transcription factors achieve coordinated regulation of sugar metabolism at multiple levels by forming complexes or interactions networks with other families of transcription factors or other proteins (Li et al., 2025). For instance, in *Glycine max*, *GmWRKY27* assembles into a complex with MYB-family transcription factor *GmMYB174*, co-repressing the expression of NAC-family factor *GmNAC29* to attenuate ABA biosynthesis while enhancing sucrose transporter *GmSWEET15* expression (Li et al., 2025). In *Vitis vinifera*, *VvWRKY22* interacts with sucrose non-fermenting-1-related kinases (*VvSnRK1.1/VvSnRK1.2*) to form a regulatory complex that phosphorylates downstream targets (*VvTPP, VvHXK*), thereby modulating glucose accumulation during cold stress (Huang et al., 2021). Furthermore, in *Prunus persica*, *PpWRKY40* physically associates with *NPR1* protein to activate *PpPRs* gene expression, while concurrently upregulating sucrose synthase (*PpSS1*) and sucrose phosphate synthase (*PpSPS3*) genes (Li et al., 2021). The WRKY structural domain of *PoWRKY69* binds directly to the VQ motif of *PoVQ11* to form a stable transcriptional regulatory complex; the *PoWRKY69-PoVQ11* module shifts the carbon flow from glycolysis to fructose synthesis through the activation of *PoFBA5* (fructose-1,6-bisphosphate aldolase gene), while inhibiting the sucrose synthase (SUS) activity and decreasing the sucrose consumption. thereby specifically elevating fructose levels (Luan et al., 2024).

### 5.2 The WRKY TFs respond to abiotic stress by regulating the glucose metabolism pathway

WRKY TFs, as an influential class of transcriptional regulators in plants, play a crucial role in plant responses to various environmental stresses. Recent studies have shown that the deep involvement of WRKY transcription factors in plant stress response is largely realized through the precise regulation of multiple pathways of sugar metabolism.

#### 5.2.1 Drought stress

Drought stress seriously threatens plant growth, development and viability. Under water deficit conditions, water absorption by the plant root system is blocked and leaf stomata are closed to reduce water loss. At the same time, cell dehydration destroys membrane structural integrity and reactive oxygen species (ROS) accumulate in large quantities, accelerating cellular senescence and even cell death. WRKY transcription factors are able to target sugar metabolism-related genes through different pathways, coordinate sugar transport, synthesis and utilization, and enhance osmoprotection and ROS scavenging, thus improving plant drought resistance. For instance, in *Paeonia ostii*, the *PoWRKY69-PoVQ11* transcription complex directly activates the key sugar metabolism gene *PoFBA5* (fructose-1,6-bisphosphate aldolase), promoting efficient fructose accumulation via its reverse catalytic function (Luan et al., 2024). The accumulated fructose exerts dual core roles: as an osmolyte to reduce cellular osmotic potential and maintain water balance, and by activating the antioxidant enzyme system while directly quenching ROS to mitigate membrane lipid peroxidation damage (Luan et al., 2024). This physiologic defense network mediated by sugar metabolism ultimately confers significant enhancement of plant drought tolerance, confirming that *PoFBA5* serves as an indispensable metabolic hub converting WRKY transcriptional regulation into drought-resistant phenotypes (Luan et al., 2024). In *Oryza sativa*, *OsWRKY11* acts as a molecular switch, specifically activating the expression of the raffinose synthase gene to promote synthesis of raffinose precursors, leading to specific raffinose accumulation in leaves (Wu et al., 2009). The accumulated raffinose functions as an efficient osmolyte to significantly decrease cellular osmotic potential, maintain cell turgor and water balance, and mitigate oxidative damage by inhibiting ROS burst. This osmotic-antioxidant collaborative network mediated by sugar metabolism translates WRKY transcriptional regulation signals into cellular homeostasis protection, ultimately endowing plants with drought-resistant phenotypes.​Under dehydration stress, the ABA signaling pathway rapidly activates *BhWRKY1* transcription factor in *Boea hygrometrica* (Wang et al., 2009). This factor precisely recognizes and binds to the W-box cis-element in the *BhGolS1* promoter, directly driving *BhGolS1* transcription. With significantly enhanced *BhGolS1* activity, massive galactinol accumulates, triggering synthesis of raffinose and stachyose, which together form key osmoprotectants (RFOs) (Wang et al., 2009). These RFOs exert drought resistance through multiple mechanisms: decreasing cellular osmotic potential and activating antioxidant enzyme systems, while promoting hydrogen bond interactions with phospholipid bilayers and membrane proteins to effectively resist dehydration-induced cell damage. Phenotypically, this results in improved cell viability, enhanced membrane structural integrity, and significantly optimized plant recovery capacity after rewatering. However, the regulatory mechanisms of WRKY transcription factors in response to drought stress show remarkable diversity. In addition to positively mediated mechanisms, some members negatively regulate sugar metabolism through distinct molecular pathways. Under drought stress, activation of *AtWRKY53* specifically binds to the promoter W-box elements of the starch-degrading gene *QQS* and the antioxidant genes *CAT2/CAT3*, initiating reprogramming of sugar metabolism. Overexpression of *QQS* accelerates the hydrolysis of starch to soluble sugars, which promotes the accumulation of malic acid via the glycolytic pathway (Sun and Yu, 2015). Malic acid, as an osmotic substance, acted synergistically with potassium ions to regulate the osmotic potential and expansion pressure of defense cells, while the attenuation of ROS signaling blocked the stomatal closure cascade, which ultimately led to an abnormal increase in stomatal conductance, an increase in transpirational loss of water, and a significant reduction in drought tolerance. This coexistence of positive and negative regulatory mechanisms reveals that the sugar metabolism pathway, as a core effector, plays a key pivotal role in connecting the molecular transcriptional network with drought stress response under the differential regulation of WRKY transcription factors.

#### 5.2.2 Salt stress

Salt stress on plants mainly manifests the triple effects of osmotic stress, ionic toxicity and oxidative damage. High-salt environments lead to elevated soil osmotic pressure, which prevents water uptake by the root system and causes drought-like physiological dehydration. Under salt stress-induced ion toxicity and osmotic stress, WRKY TFs alleviate osmotic imbalance by triggering starch-to-soluble sugar conversion. They orchestrate synergistic coordination between sugar signaling and ion transport systems (e.g., Na^+^/H^+^ antiporters), thereby reinforcing membrane integrity. Concurrently, sugar-derived metabolites modulate antioxidant defenses to mitigate oxidative injury. For instance, under salt stress, *VvWRKY30* drives remodeling of sugar metabolism by specifically activating sugar transport genes (*VvHT1/5*) and metabolic genes (*VvSS, VvHXK, VvTRE*). On one hand, it promotes the accumulation of glucose, fructose, and trehalose as osmotic regulators to lower cellular osmotic potential and maintain turgor pressure. On the other hand, it enhances the pentose phosphate pathway to supply NADPH, which boosts the activities of antioxidant enzymes like superoxide dismutase (SOD), peroxidase (POD), and catalase (CAT) – effectively scavenging H_2_O_2_ and reducing membrane lipid peroxidation (Cao et al., 2025b). Additionally, HXK-mediated sugar signaling amplifies stress responses in a cascading manner, forming a positive feedback loop with ethylene signaling. This ultimately maintains photosynthetic function, safeguards reproductive development, and reduces biomass loss at the phenotypic level, achieving an integrated salt-tolerance mechanism from transcriptional regulation to physiological adaptation (Zhu et al., 2019). In *Rosa rugosa*, *RrWRKY1* maintains cellular osmotic balance by regulating proline accumulation under salt stress (Zang et al., 2024). As an intermediate metabolite in sugar metabolism, proline is generated from glutamate via the glycolytic pathway. Experiments show that silencing *RrWRKY1* leads to a significant decrease in proline content and an increase in malondialdehyde (MDA) content, indicating that this transcription factor enhances antioxidant capacity through sugar metabolism-related pathways to alleviate oxidative damage caused by salt stress (Zang et al., 2024).​ In conclusion, WRKY transcription factors regulate sugar metabolic pathways to not only cope with osmotic stress and ion toxicity from salt stress but also enhance plant antioxidant defense capabilities. This establishes multilayered salt-tolerance mechanisms spanning from gene expression regulation to physiological function adaptation, providing crucial molecular guarantees for plant survival in high-salt environments.

#### 5.2.3 Cold stress

WRKY transcription factors play a central role in plant responses to low-temperature stress by dynamically regulating sugar metabolic pathways. Under low-temperature conditions, which disrupt membrane fluidity and inhibit photosynthesis, plants maintain osmotic balance and energy supply through the accumulation of soluble sugars. Members of the WRKY family activate the expression of key enzymes such as amylase and sucrose synthase, thereby promoting sugar accumulation and enhancing cold tolerance. Take the cold-inducible WRKY transcription factor *CdWRKY2* as an example (Huang et al., 2022b). It directly binds to the W-box elements in the promoter regions of the sucrose phosphate synthase gene (*CdSPS1*) and the *CBF1* gene, activating their transcriptional expression (Huang et al., 2022b). The product of *CdSPS1*, serving as both an osmolyte and a signaling molecule, exerts dual regulatory functions: it enhances cellular osmotic homeostasis by accumulating sucrose, which lowers the freezing point and maintains membrane integrity; meanwhile, it activates the pentose phosphate pathway to generate NADPH, thereby improving cellular antioxidant capacity. Additionally, *CdWRKY2* collaborates with the core gene *CdCBF1* of the CBF signaling pathway to coordinately regulate the expression of downstream cold-responsive genes. The synergistic action of these two regulatory pathways ultimately enhances the tolerance of transgenic Arabidopsis to low-temperature stress significantly. In *Raphanus sativus*, the *RsWRKY40* transcription factor acts as a central regulatory hub to coordinate cold resistance mechanisms through a dual-function mode (Chen et al., 2025). It not only directly activates the sucrose phosphate synthase gene *RsSPS1* to promote sucrose accumulation for osmotic protection and energy supply but also simultaneously induces the CBF signaling pathway to activate downstream antifreeze genes. Sucrose serves as a critical bridging molecule in this process: it functions as a protective metabolite to maintain membrane stability and reactive oxygen species (ROS) scavenging, while also reinforcing the CBF pathway and its own synthesis through feedback regulation. Research in *Camellia sinensis* has shown that *CsWRKY29* activates sugar metabolic genes such as sucrose phosphate synthase (*CsSPS1*) and hexokinase (*CsHXK1*) under low temperature, promoting the synthesis and accumulation of sucrose and hexoses (Xue et al., 2024). This transcription factor orchestrates two parallel processes: one involves activating sucrose degradation and glycolysis to ensure adenosine triphosphate (ATP) supply, and the other promotes the synthesis of osmoprotective oligosaccharides (trehalose, raffinose) and flavonoid glycosides. The former maintains cellular osmotic balance and membrane stability, while the latter enhances antioxidant activity through glycosylation modification. Furthermore, *CsWRKY29* strengthens the expression of sugar metabolic genes via the abscisic acid (ABA) signaling pathway, forming a “ABA-*CsWRKY29*-Sugar metabolism” positive feedback loop (Xue et al., 2024). It also synergistically activates the CBF-COR pathway, integrating sugar metabolism with antifreeze protein synthesis to achieve enhanced freezing tolerance through multi-pathway coordination. Beyond endogenous regulatory mechanisms, WRKY transcription factors may also mediate low-temperature stress responses through exogenous sugar application. For instance, exogenous sucrose supplementation compensates for the insufficient sucrose synthesis caused by *RsWRKY40* silencing, indirectly demonstrating that exogenous sugars alleviate cold damage by regulating the *RsWRKY40*-mediated sugar metabolic network (Chen et al., 2025). In *Cucumis sativus*, treatment with exogenous trehalose significantly upregulates WRKY gene expression, induces soluble sugar synthesis, and thereby mitigates cold injury (Pan et al., 2022).

In summary, WRKY transcription factors can precisely regulate the metabolism of sugars by utilizing the “WRKY - Sugar Metabolism” module in order to participate in plant stress response, and this module plays a key role in drought, salt, low temperature, and a variety of abiotic stresses, which provides an important survival and reproduction of plants in harsh natural environments. However, significant gaps remain in understanding WRKY-mediated thermotolerance (Zhang et al., 2024b). Limited studies suggest WRKYs participate in heat stress by regulating anti-oxidant systems and membrane stability, but whether this involves sugar metabolism remains elusive, presenting a key avenue for future research (Dang et al., 2013).

## 6 Conclusions and perspectives

WRKY transcription factors are ubiquitously distributed across the plant kingdom and play pivotal roles in regulating plant growth, developmental programs, and stress-responsive mechanisms. In recent years, a growing body of research has focused on the mediation of abiotic stress by WRKY transcription factors through sugar metabolic pathways, with remarkable advancements achieved in research methodologies. Notwithstanding these progresses, this field still harbors significant prospects and unresolved research gaps that warrant systematic exploration.

First and foremost, future investigations should endeavor to elucidate in greater detail the specific molecular mechanisms underlying the interaction between WRKY transcription factors and sugar metabolic pathways. Although existing evidence has established that WRKY transcription factors modulate the expression of sugar metabolism-related genes, the intricate regulatory networks and precise target sites thereof remain incompletely characterized. To address this, future studies are expected to employ cutting-edge technical approaches, including gene-editing technologies (particularly multi-gene editing to circumvent functional redundancy) (Cao et al., 2025a), high-resolution protein-protein interaction analyses (such as *in vivo* co-immunoprecipitation, yeast two-hybrid library screening, and proximity labeling techniques) (Cao et al., 2024), and single-cell omics. These methodologies will facilitate the precise dissection of how WRKY proteins recognize and bind to the promoter regions of downstream sugar metabolism genes, as well as how their complexes with other transcription factors or coregulatory molecules exert fine-scale regulation over the activity of key enzymes, thereby influencing the dynamic homeostasis of critical sugar molecules.

Second, it is imperative to resolve the long-standing challenges of functional redundancy and specificity within the WRKY gene family. Comprising a large repertoire of members, the WRKY family often exhibits extensive functional redundancy or overlap, rendering traditional genetic approaches inadequate for accurately evaluating the contribution of individual members in sugar metabolism-stress response cascades. Moreover, distinct WRKY members may exhibit context-dependent functions under varying stress conditions, in different tissues/organs, or at specific developmental stages. Future research should integrate systems biology approaches with conditional gene-editing/inducible expression systems to meticulously dissect the specific roles of different WRKY members in regulating sugar metabolism under defined environmental and physiological contexts, along with the underlying molecular determinants.

Furthermore, more direct physiological and metabolic evidence is required to establish the causal relationship between WRKY-mediated sugar metabolism and enhanced stress tolerance. Current investigations predominantly focus on molecular-level analyses or terminal phenotypic observations, whereas the evidentiary chain for intermediate links remains fragmented. Specifically, following the regulation of specific sugar metabolism genes by WRKY, how do changes in sugar composition, concentration, spatiotemporal distribution, and cellular energy status directly impact key physiological processes, including stress signal perception, reactive oxygen species scavenging, osmotic adjustment, and maintenance of cell membrane integrity? Future studies should integrate metabolomics, enzyme activity assays, subcellular localization analyses, and live-cell imaging technologies to track, at high spatiotemporal resolution, how WRKY-mediated reprogramming of sugar metabolism translates into specific physiological and biochemical responses that underpin plant stress resilience.

In conclusion, the field of WRKY transcription factors mediating abiotic stress through sugar metabolic pathways presents substantial scope for advancement. Future research should prioritize the in-depth investigation of regulatory mechanisms, expansion of research frontiers, and comprehensive consideration of their roles across multiple metabolic networks, thereby fostering a holistic understanding of WRKY transcription factors in plant stress adaptation. Such endeavors are critical for the breeding of stress-tolerant crop varieties and the promotion of sustainable development in China’s forestry and agricultural sectors.
