# Supplementary material for: WRKY transcription factors participate in abiotic stress responses mediated by sugar metabolism
Source: Front Plant Sci. 2025 Aug 7;16:1646357. doi: 10.3389/fpls.2025.1646357 (PMC12367783; doi:10.3389/fpls.2025.1646357)
Supplement: Supplementary file 1 [file Presentation1.zip › Frontiers_Supplementary_Material/Supplementary_Material.docx]

Supplementary Material

# Supplementary Figures and Tables

## Supplementary Figures

**
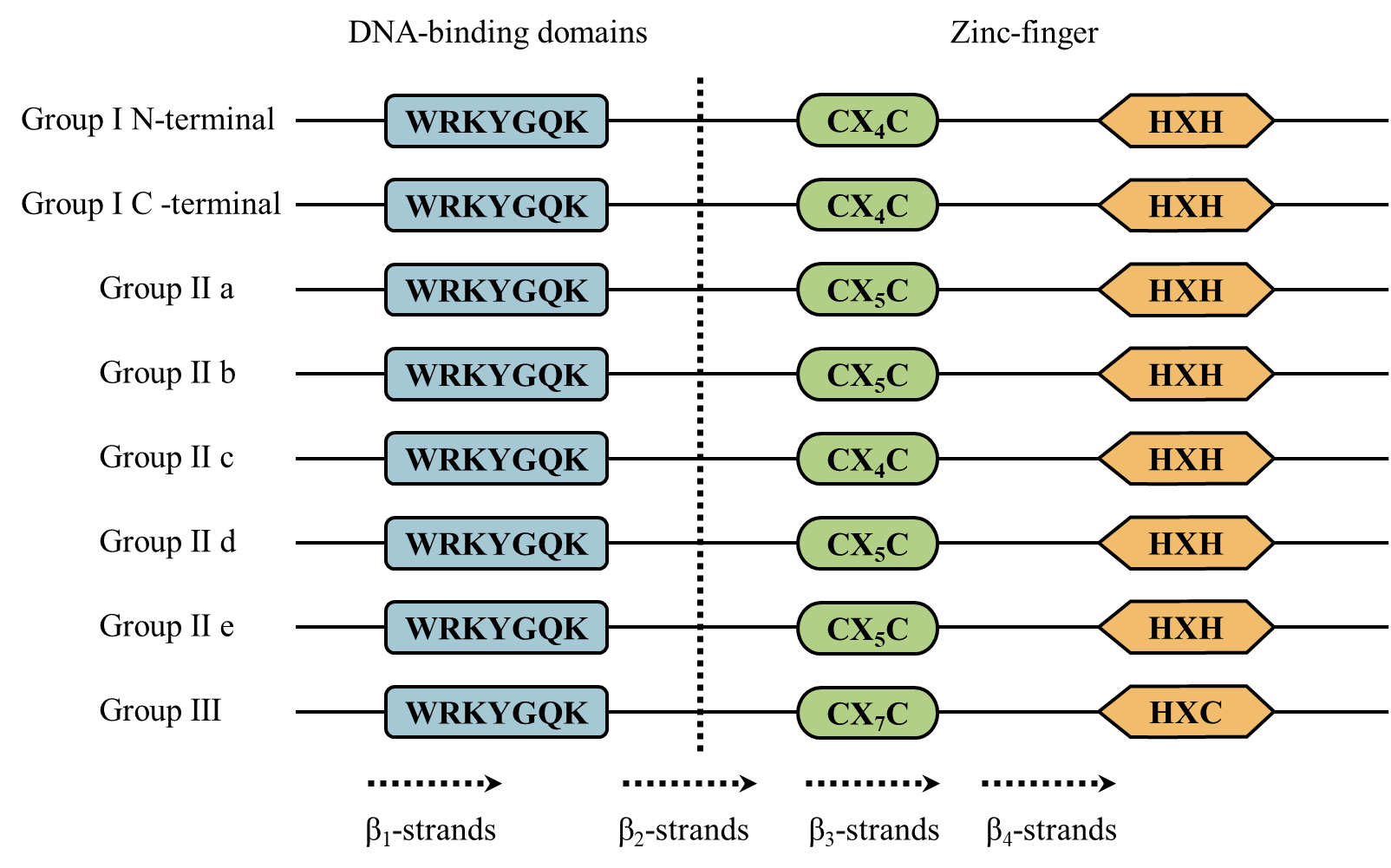
**

**Supplementary Figure 1**: Domain structures of different WRKY subfamilies in higher plants. The WRKY motif, the cysteines, and the histidines that form the zinc finger are shown in boxes. I N and I C denote the N-terminal and C-terminal domains from Group I WRKY proteins, respectively. The 4 β-strands are shown with dashed arrows.


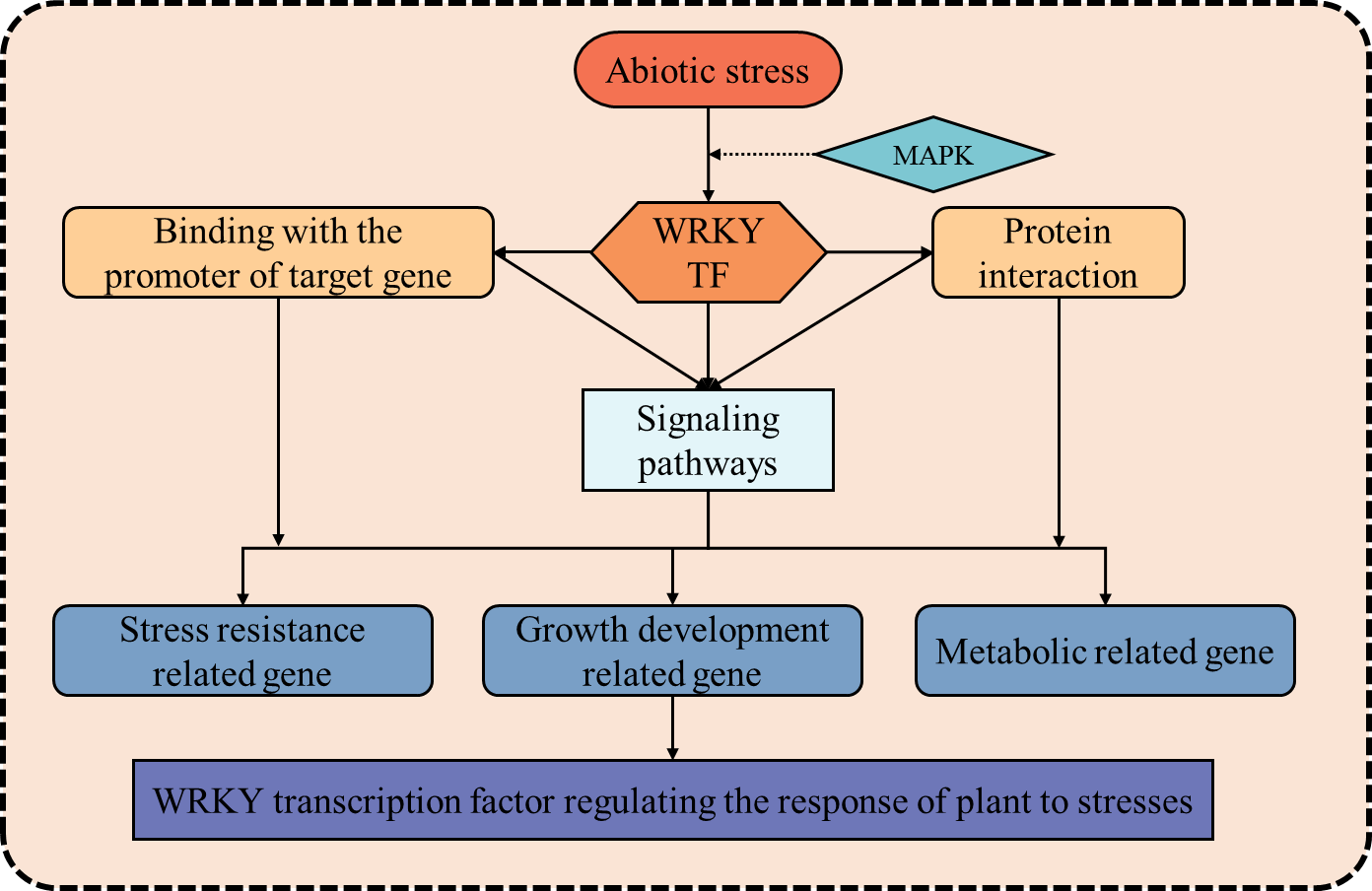


**Supplementary Figure 2：**The diagram of WRKY transcription factor regulating stress responses in plants. The solid black arrows indicate that WRKYs regulating plant stress response pathway; The dotted black arrow indicates that WRKY transcription factors might be activated by the MAPK cascade and thus participates in the regulation of stress response.


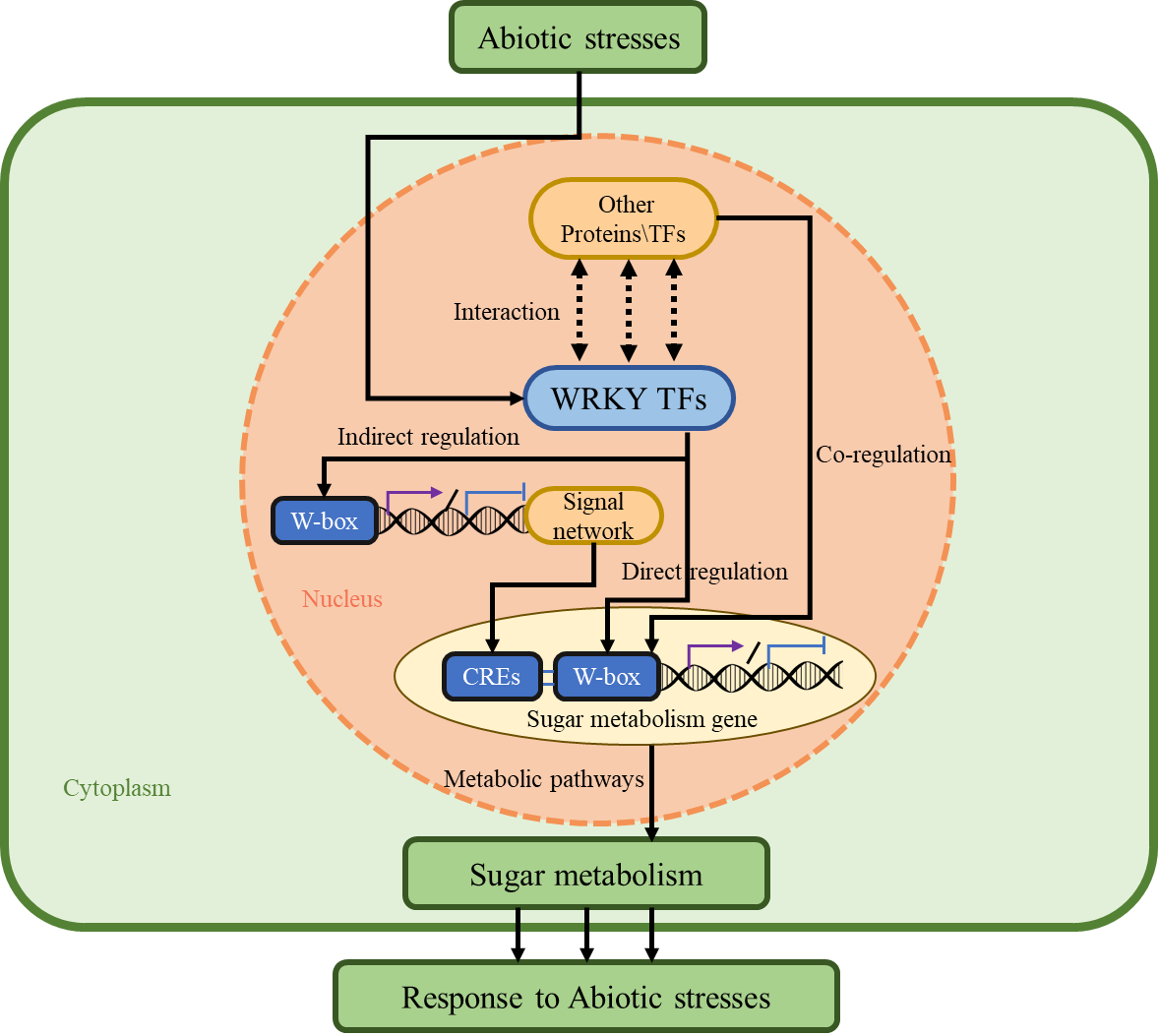


**Supplementary Figure 3：**The mechanism by which WRKY TFs regulate glucose metabolism and mediate abiotic stress.

## Supplementary Tables

**Supplementary Tables 1.** WRKY TFs involved in abiotic stress responses in plants.

| **No.** | ***Gene*** | ***Species*** | **Stress responses** | **References** |
| --- | --- | --- | --- | --- |
| 1 | *MaWRKY70* | *Musa acuminata* | Tolerance to cold | (Lin et al., 2024) |
| 2 | *OsWRKY63* | *Oryza sativa* | Tolerance to cold | (Zhang et al., 2022b) |
| 3 | *OsWRKY76* | *Oryza sativa* | Tolerance to cold | (Naoki et al., 2013) |
| 4 | *OsWRKY74* | *Oryza sativa* | Tolerance to phosphate (Pi) starvation | (Dai et al., 2016) |
| 5 | *CsWRKY19* | *Camellia sinensis* | Tolerance to cold | (Guo et al., 2024) |
| 6 | *VpWRKY1* | *Vitis pseudo-reticulata* | Tolerance to salt and cold | (Li et al., 2010) |
| 7 | *VpWRKY2* | *Vitis pseudo-reticulata* | Tolerance to salt and cold | (Li et al., 2010) |
| 8 | *GmWRKY13* | *Glycine max* | Tolerance to drought and cold | (Zhou et al., 2008) |
| 9 | *GmWRKY21* | *Glycine max* | Tolerance to drought and cold | (Zhou et al., 2008) |
| 10 | *GmWRKY54* | *Glycine max* | Tolerance to drought and cold | (Zhou et al., 2008) |
| 11 | *CsWRKY51* | *Cucumis sativus* | Tolerance to cold | (Lu et al., 2025) |
| 12 | *CwWRKY65* | *Camellia weiningensis* | Tolerance to cold | (Xu and Xu, 2024) |
| 13 | *VbWRKY32* | *Verbena bonariensis* | Tolerance to cold | (Wang et al., 2020) |
| 14 | *VvWRKY24* | *Vitis vinifera* | Tolerance to cold | (Wang et al., 2014a) |
| 15 | *AtWRKY34* | *Arabidopsis* | Tolerance to cold | (Zou et al., 2010) |
| 16 | *PoWRKY69* | *Paeonia ostii* | Tolerance to drought | (Luan et al., 2024) |
| 17 | *MbWRKY46* | *Malus baccata* | Tolerance to drought and cold | (Liu et al., 2023) |
| 18 | *ChaWRKY40* | *Corylus avellana* | Enhances Drought Tolerance | (Zhang et al., 2024a) |
| 19 | *PwuWRKY48* | *Populus wulianensis* | Enhances Drought Tolerance | (Wang et al., 2024) |
| 20 | *EjWRKY17* | *Eriobotrya japonica* | Enhances Drought Tolerance | (Wang et al., 2021) |
| 21 | *IgWRKY32* | *Iris germanica* | Enhances Drought Tolerance | (Zhang et al., 2022a) |
| 22 | *IgWRKY50* | *Iris germanica* | Enhances Drought Tolerance | (Zhang et al., 2022a) |
| 23 | *PtWRKY33* | *Populus trichocarpa* | Tolerance to drought and salt | (Yang et al., 2023) |
| 24 | *ZmWRKY40* | *Zea mays* | Enhances Drought Tolerance | (Wang et al., 2018) |
| 25 | *IlWRKY70* | *Iris laevigata* | Tolerance to drought and salt | (Shi et al., 2023) |
| 26 | *BnWRKY49* | *Boehmaeria nivea* | Enhances Drought Tolerance | (Bao et al., 2024) |
| 27 | *AfWRKY2* | *Amorpha fruticosa* | Enhances Drought Tolerance | (Li et al., 2023) |
| 28 | *StWRKY6* | *Solanum tuberosum* | Tolerance to cadmium (Cd) | (He et al., 2023) |
| 29 | *OsWRKY54* | *Oryza sativa* | Tolerance to salt | (Huang et al., 2022) |
| 30 | *VuWRKY21* | *Vigna unguiculata* | Tolerance to salt | (Crispim et al., 2023) |
| 31 | *VuWRKY87* | *Vigna unguiculata* | Tolerance to salt | (Crispim et al., 2023) |
| 32 | *ZjWRKY18* | *Ziziphus jujuba* | Tolerance to salt | (Wen et al., 2023) |
| 33 | *AhWRKY75* | *Arachis hypogaea* | Tolerance to salt | (Zhu et al., 2021) |
| 34 | *GmWRKY16* | *Glycine max* | Tolerance to drought and salt | (Ma et al., 2019) |
